# Nociception and pain assessment during suctioning procedures in mechanically ventilated patients in the intensive care unit: A validation study of the Nociception Level index (NOL™)

**DOI:** 10.1080/24740527.2025.2561576

**Published:** 2026-04-29

**Authors:** Shiva Shahiri, Philippe Richebé, Marc O. Martel, Patrick Lavoie, Céline Gélinas

**Affiliations:** a Ingram School of Nursing, McGill University, Montreal, Quebec, Canada; b Centre for Nursing Research and Lady Davis Institute, Jewish General Hospital–CIUSSS West-Central-Montreal, Montreal, Quebec, Canada; c Département d’Anesthésiologie, Bordeaux Nord Aquitaine, PBNA, Bordeaux, France; d Department of Anesthesiology and Pain Medicine, University of Montreal, Montreal, Quebec, Canada; e Faculty of Dental Medicine & Department of Anesthesiology, McGill University, Montreal, Quebec, Canada; f Alan Edwards Pain Management Unit, McGill University Health Centre, Montreal, QC, Canada; g Faculty of Nursing, Université de Montréal, Montreal, Quebec, Canada; h Montreal Heart Institute Research Center, Montreal, Quebec, Canada

**Keywords:** NOL index, pain assessment, nociception, intensive care unit, validity, reliability, heart rate

## Abstract

**Background:**

Many patients in the intensive care unit (ICU) cannot communicate pain through self-reports or behaviors. Though individual physiologic parameters (e.g. heart rate, HR) lack validity for ICU nociception and pain assessment, a multiparameter approach (i.e. Nociception Level index, NOL) has shown promise in anesthesia, but its use in the ICU is new.

**Aim:**

The aim of this study was to validate the NOL for ICU nociception and pain assessment in mechanically ventilated patients.

**Methods:**

In this prospective observational study, NOL values (0–100) were recorded before, during, and 15 min after a nonnociceptive procedure (blood pressure cuff inflation) and a nociceptive procedure (mouth, endotracheal, or tracheal suctioning) in patients able or not to self-report. Validation included discriminative (nociceptive vs. nonnociceptive procedures), criterion (pain intensity and Critical Care Pain Observation Tool, CPOT) and convergent (procedural pain distress) strategies, and test–retest reliability. HR validity was also examined.

**Results:**

Of 53 enrolled patients, we had 14 losses (9 NOL-related), thus data from 39 were analyzed. Missing data occurred in 25% of patients at some time points. NOL median values were significantly higher during suctioning (>25) than cuff inflation (<10) and remained stable pre/postsuctioning. Positive correlations were found for pain intensity and CPOT but not for procedural pain distress. HR increased slightly during suctioning but was not correlated with pain criteria.

**Conclusions:**

The discriminative validity of the NOL was supported for ICU nociception in all patients and showed stable resting values. Criterion validity for pain assessment was only significant in patients able to self-report. Convergent validity was not supported. HR showed poor validity.

## RÉSUMÉ

**Abrégé Contexte:** Plusieurs patients à l’unité des soins intensifs (USI) sont incapables de communiquer leur douleur par l’autoévaluation ou par des comportements observables. Bien que certains paramètres physiologiques individuels, comme la fréquence cardiaque (FC), ne sont pas valides pour l’évaluation de la nociception et de la douleur à l’USI, une approche multiparamétrique (NOL pour Nociception Level Index) a montré des résultats prometteurs en anesthésie, mais son utilisation à l’USI est nouvelle.

**Objectif:** Valider le NOL pour l’évaluation de la nociception et de la douleur à l’USI chez des patients sous ventilation mécanique.

**Méthodes:** Dans cette étude observationnelle prospective, les valeurs du NOL (0–100) ont été obtenues avant, pendant et 15 minutes après une procédure non nociceptive (gonflage du brassard pour la prise de pression artérielle) et une procédure nociceptive (aspiration buccale, endotrachéale ou trachéale) chez des patients capables ou non de s’autoévaluer. La validation comprenait des stratégies de validité discriminative (procédures nociceptives vs non nociceptives), de validité de critère (perception d’intensité de la douleur et observation de comportements [CPOT]) et de validité convergente (détresse liée à la douleur procédurale), ainsi qu’une analyse test–retest (stabilité). La validité de la FC a également été examinée.

**Résultats:** Parmi les 53 patients recrutés, des pertes (n=14) sont survenues dont neuf liées au NOL, de sorte que les données de 39 patients ont été analysées. Des données manquantes ont été observées chez 25 % des patients à certains moments. Les médianes du NOL étaient significativement plus élevées pendant l’aspiration (> 25) que lors du gonflage du brassard (< 10) et sont demeurées stables avant et après l’aspiration. Des corrélations positives ont été observées pour les scores d’intensité de la douleur et du CPOT, mais non pour la détresse liée à la douleur procédurale. La FC a légèrement augmenté pendant l’aspiration, sans association avec les critères de douleur.

**Conclusions:** La validité discriminative du NOL a été confirmée pour la nociception à l’USI et les valeurs au repos sont demeurées stables. La validité de critère s’est montrée significative seulement chez les patients capables de s’autoévaluer. Les résultats n’ont pas soutenu la validité convergente. La FC a démontré une faible validité.

## Introduction

Pain is a common symptom in the intensive care unit (ICU), affecting critically ill adults.^1^ It primarily arises from nociception caused by tissue damage, which may result from underlying illnesses, trauma, surgery, invasive interventions (e.g., mechanical ventilation), or noxious stimuli associated with standard care procedures (e.g., turning, endotracheal suctioning, catheter insertion/removal).^2–6^ Though critically ill adults experience pain at rest, the intensity of their pain increases significantly during standard care procedures.^2–7^

Proper and routine pain assessment in the ICU should be performed in all critically ill adults.^1^ Indeed, acute pain has been associated with severe adverse events (e.g., tachycardia, oxygen desaturation)^8^ and is a major risk factor for chronic pain development in critically ill survivors.^9–11^ Therefore, adequate pain assessment is vital to guide informed decisions on pain management in ICU patients.^12^

Nociception and pain are distinct yet interrelated concepts. Nociception refers to the physiologic neural process of encoding a noxious stimulus that damages or threatens damage to normal tissue^13^ and that may lead to pain sensation. Pain is a multidimensional concept described as “an unpleasant sensory and emotional experience associated with, or resembling that associated with, actual or potential tissue damage.”^14(p1977)^ Although the inability to communicate verbally does not negate the experience of pain, self-report should be respected as pain is a personal experience.^13^ Accordingly, the patient’s self-report serves as the reference standard measure of pain.^1,13^ However, many ICU patients are unable to self-report due to their critical care condition and related treatment (e.g., mechanical ventilation, use of sedative or neuromuscular blocking agents) altering their level of consciousness and capacity to communicate through self-reporting.^1,15^

Pain encompasses not only sensory and emotional dimensions but also behavioral and physiologic responses.^16,17^ When self-reporting is impossible to obtain, practice guidelines^1^ and recommendations^15^ suggest using alternative behavioral standard measures of pain such as the Critical Care Pain Observation Tool (CPOT^18^) or the Behavioral Pain Scale.^19^ Nonetheless, pain behaviors can be masked in patients who are heavily sedated or suffering from severe neurological damages affecting their motor system to exhibit behavioral reactions to noxious stimuli.^1,15,20^

When neither self-reporting nor behavioral responses are available, ICU nurses lack a valid measure for pain assessment. In such cases, individual vital signs such as heart rate (HR) and blood pressure (BP) monitored continuously at the ICU bedside appear to be the only alternatives readily available. However, evidence has shown that these individual parameters are not valid for ICU pain assessment.^1,15,21^ Interestingly, the simultaneous combination of multiple physiologic parameters has been shown to be superior to their individual use for the assessment of nociception and pain in healthy adults^22^ and anesthetized mechanically ventilated adults.^23–27^

To our knowledge, the Nociception Level index (NOL)^28^ is the sole technology that has been designed to incorporate several physiologic parameters simultaneously to estimate nociception level during anesthesia.^23^ NOL values increased significantly, by a median ranging from 16 to 39 points, following nociceptive stimuli (i.e., an actual or potentially tissue-damaging event transduced and encoded by nociceptors^13^) such as intubation, skin incision, or tetanic stimulation, compared to resting periods.^29^ Comparisons of the NOL to individual physiological parameters (e.g., HR, BP, skin conductance) further demonstrated its superior performance, with area under the curve values consistently >0.90 for the NOL versus <0.70 for single parameters.^29^ Collectively, these findings support the NOL’s robust discriminative ability and its advantage as a multiparameter technology for nociception assessment under general anesthesia; however, its use in the ICU is new. Thus, we conducted the first pilot studies evaluating the NOL for nociception and pain assessment in conscious ICU patients (mechanically ventilated or not) able to self-report their pain during chest tube removal and endotracheal suctioning.^30,31^ Our findings suggested that the NOL could discriminate between nociceptive and nonnociceptive ICU procedures and was associated with ICU pain reference and alternative standards. Moreover, our findings supported the feasibility of the research methods and procedures with a few challenges associated with using the NOL in the ICU (e.g., signal losses). Although mitigation strategies were implemented to improve NOL signal issues, these highlighted the need for further evaluation and optimization of the device in the complex ICU environment. Additionally, our pilot studies with NOL were conducted exclusively in patients who were able to self-report pain; therefore, further feasibility monitoring is warranted in patients who cannot self-report and further research is needed to support the validity of the NOL for ICU nociception and pain assessment.

### Objectives

This study aimed to validate the NOL for nociception and pain assessment in mechanically ventilated ICU adults able or not to self-report their pain based on their level of consciousness. We also explored the performance of HR for ICU nociception and pain assessment, because HR is one of the most commonly used vital signs for pain assessment,^21^ and the NOL device generates HR values in its output, ensuring consistency in measurement of both parameters.

The specific study objectives were to examine:

1. Discriminative validation: The NOL’s ability to discriminate between nonnociceptive and nociceptive procedures in mechanically ventilated ICU patients able or not to self-report according to their level of consciousness.
2. Criterion validation: The NOL’s ability to detect pain using the reference standard measure of pain (i.e., self-reported pain intensity) or an alternative behavioral standard measure (i.e., CPOT), according to patients’ capacity to communicate.
3. Convergent validation: The NOL’s association with another theoretically related construct or dimension (i.e., emotional dimension of pain) using self-reported procedural pain distress in mechanically ventilated ICU patients able to self-report.
4. Test–retest reliability: The NOL’s ability to yield the same values over time in similar conditions (i.e., before and after a nociceptive procedure).

As secondary objectives, the same validation strategies were explored for HR, and the feasibility of the research methods for the NOL validation is described to inform future clinical and research applications.

## Materials and methods

### Study design

A prospective observational design, including repeated measures within subjects, was appropriate for this validation study.^32^ The STROBE (Strengthening the Reporting of Observational Studies in Epidemiology) checklist was used for the reporting of the study methods and findings.^33^

### Setting

The study was conducted from July 2022 to March 2023 in the medical–surgical ICU of a large tertiary metropolitan university-affiliated hospital in Montreal, Canada. This ICU has 30 beds. However, bed capacity was reduced during the study to 20 due to staffing shortages arising from the COVID-19 pandemic, with an annual average of 1000 to 1500 ICU admissions.

### Participants

Through a consecutive sampling method, we recruited patients admitted to the ICU who were mechanically ventilated and assigned them to group A or group B according to their level of consciousness (LOC) and capacity to communicate:

Group A: Conscious (Glasgow Coma Scale^34^ = 13–15 and/or Richmond Agitation–Sedation Scale [RASS]^35^ = 0), able to self-report their pain (by head nodding or pointing to scales) and to exhibit pain behaviors.

Group B: Altered LOC (Glasgow Coma Scale ≤ 12 and/or RASS ≤ −1) and unable to self-report but might exhibit pain behaviors.

Including varying levels of consciousness enabled the use of self-reported pain intensity for conscious individuals and the use of an alternative behavioral standard for both groups.

Patients meeting the following inclusion criteria were eligible: (1) 18 years or older, (2) English or French speaking, and (3) mouth, endotracheal, or tracheal suctioning as part of the patient’s standard care. Patients were excluded under these conditions: (1) absence of an accessible finger for the installation of the NOL’s finger probe, (2) severe peripheral vascular disease affecting the upper limbs, (3) cardiac arrhythmia impacting heart rate variability, (4) pacemaker, (5) hypoperfusion state or shock necessitating high doses of norepinephrine (>14 mcg/min) or equivalent vasopressors inducing vasoconstriction,^36^ (6) agitation (RASS = +1 to +4), (7) cognitive deficits (e.g., Alzheimer’s) or psychiatric conditions (e.g., psychosis) as documented in the patient’s medical chart, (8) pregnancy, or, (9) in group A, positive on delirium screening at the time of data collection. These exclusion criteria were identified to account for potential confounding factors that could impact the generation or quality of the NOL signal, thus enhancing the study’s internal validity. Specifically, conditions affecting HR, BP, and hand perfusion may lead to incapacity to generate a NOL signal or signal losses. Furthermore, the NOL probe is susceptible to signal loss due to excessive movements caused by agitation. Lastly, delirium can compromise reliable self-reporting, and behavioral responses may be altered in individuals with cognitive or psychiatric conditions.

## Data collection

### Instruments and variables

This study included five variables (i.e., NOL, HR, pain intensity, CPOT, and procedural pain distress). The NOL was the primary variable of this validation study.

#### Nociception Level Index and HR

The NOL, provided by the PMD-200 monitor (Medasense Biometrics Ltd.), is derived from multiple physiologic parameters that are extracted simultaneously, including HR, HR variability, photoplethysmography pulse wave amplitude, skin conductance and its fluctuation, skin temperature, and their time derivatives.^23^ The NOL ranges from 0 to 100 and values greater than 20 to 25 have been identified to be indicative of nociception and pain.^26,31,37–39^ Preliminary findings of the NOL validation testing in the ICU supported discriminative and criterion validity with pain reference and alternative standards in mechanically ventilated patients able to self-report (*n* = 15) and postoperative cardiac patients (*n* = 54).^30,31^ NOL values were increased during nociceptive procedures (i.e., endotracheal suctioning, chest tube removal) compared to rest and a nonnociceptive procedure (i.e., noninvasive blood pressure, NIBP).^30,31^ NOL values correlated with self-reported pain intensity and CPOT scores.^30,31^ A NOL cutoff >25 adequately identified ICU patients with moderate to severe self-reported pain intensity.^31^ HR is the only single parameter directly available for offline extraction from the device. Consequently, HR values were extracted in parallel with the NOL index for analysis.

#### Pain intensity—reference standard measure of pain

The 0–10 Facial Pain Thermometer (FPT) is graded from 0 = *no pain* to 10 = *worst possible pain* in a visual thermometer format, which also includes six faces.^40^ The FPT has shown convergent validity in cardiac surgery ICU patients with a 4-point descriptive pain scale (*r* = 0.80–0.86, *P* < 0.001), discriminative validity between rest and turning (*t*-test, *P* < 0.001), and content validity.^40^ The FPT was also successfully used in our NOL pilot studies and other CPOT validation studies with surgical, medical, and trauma ICU patients.^30,31,41^

#### Critical-Care Pain Observation Tool—alternative standard measure of pain

The CPOT is a behavioral tool developed for pain assessment in ICU patients unable to self-report. It includes four behavioral items: (1) facial expression, (2) body movements, (3) muscle tension, and (4) compliance with the ventilator (for mechanically ventilated patients) or vocalization (for non–mechanically ventilated patients). Each item is rated on a scale from 0 to 2, yielding a total score ranging from 0 to 8:

- Facial expression: Score 0 = relaxed face with no muscle tension; score 1 = tense face, often shown by brow lowering; score 2 = grimacing, with full-face contraction (eyes tightly closed, cheek muscle contraction, possible mouth opening, or biting the endotracheal tube).
- Body movements: Score 0 = no movement or normal position; score 1 = protective movements, such as slow, cautious attempts to touch the pain site; score 2 = restlessness or agitation, including repetitive movements, pulling at tubes, or attempting to sit up.
- Compliance with the ventilator (mechanically ventilated patients): Score 0 = easy, synchronous breathing without alarms; score 1 = occasional alarms resolving without intervention; score 2 = asynchrony requiring clinical intervention.
- Vocalization (nonintubated patients): Score 0 = no sound or normal speech; score 1 = sighing or moaning; score 2 = crying out or sobbing.
- Muscle tension: Assessed by passive movement of the arm (at rest) or during turning. Score 0 = no resistance; score 1 = resistance indicating tension or rigidity; score 2 = strong resistance, with the patient resisting movements or clenching fists.

Patients are observed over a 1-min period when at rest or for the time of the procedure to detect behaviors. The research trainee and research staff were trained to use CPOT via an educational session created by the tool developer^42^ and used the CPOT in NOL pilot studies.^30,31^ CPOT has been largely validated in more than 20 countries^30,31^ and has been largely validated in more than 20 countries, with over 3900 ICU patients admitted for various diagnoses.^41^ It has demonstrated good interrater reliability (intraclass correlation coefficient [ICC] ≥ 0.60 and percentage agreement ≥80 between research staff and ICU nurses), discriminative validity between nociceptive (e.g., turning/repositioning, mouth, endotracheal and tracheal suctioning) and nonnociceptive procedures (e.g., NIBP, soft touch), and criterion validity (*r* > 0.40) with self-reported pain intensity.^41^ A CPOT cutoff score ≥ 3 has been established to adequately detect the presence of pain with an area under the curve range of 0.72 to 0.91.^41,43^

#### Procedural pain distress self-report scale

The 0–10 numeric rating scale (NRS) was used to assess the procedural pain distress experienced by patients during suctioning procedures, which relates to the emotional dimension of procedural pain.^44^ It is scored on a visual numeric scale from 0 = *not at all distressful* to 10 = *most distressful feeling possible*. This scale has shown convergent validity with the 0−10 NRS pain intensity during ICU nociceptive procedures (*r* = 0.79, *P* < 0.001) in a large multicenter study (192 ICUs in 28 countries) including 3851 critically ill adults.^45^

#### Demographic and clinical data

Demographic data, including age, sex, gender, ethnicity, language most spoken at home, and education level, were obtained from participants or their family members. Clinical information extracted from their medical chart included diagnosis, Acute Physiology and Chronic Health Evaluation II (APACHE II) score,^46^ sedation level (RASS),^35^ delirium assessment (Confusion Assessment Method–ICU),^37^ and some medications (i.e., opioids, sedatives, neuromuscular blocking agents, and vasopressors). Opioid doses received within 4 h before and during data collection were converted into oral morphine-equivalent doses (mg).^47^

#### Feasibility of research methods

Feasibility pertains to the ease or convenience of the execution of research methods^48^ including (1) sufficiency of the sampling pool (participant flow diagram), (2) simplicity of participant screening process, (3) efficiency of recruitment time (i.e., number of eligible and enrolled patients) during the study period, and (4) ease of data collection (i.e., issues with the NOL’s use and mitigation strategies).

### Procedures

The research project was submitted to the Medical and Biomedical Research Ethics Committee in February 2022 and obtained approval in June 2022 (Project No. MP-05-2022-2988). Subsequently, an amendment was approved to remove one of the eligibility criteria, the requirement for a minimum 24-h ICU stay, to facilitate participant recruitment.

ICU patients were screened for eligibility by the research trainee in collaboration with ICU nurses. The eligible ICU patients who were able to self-report and provided written consent were assigned to group A upon agreeing to participate. Those unable to self-report or consent were assigned to group B. Given that this study involved only minimal risk, a mandatory or a person qualified to provide consent for health care decisions was asked to provide consent on their behalf. When a patient participant who could not previously consent regained this ability later during their hospitalization, the research trainee provided them with the informed consent form, enabling them to decide on their continued participation.

Before data collection began, the research trainee confirmed the participant’s group assignment. For group A and to ensure reliable self-reporting, the research trainee verified delirium status (an exclusion criterion) by checking the Confusion Assessment Method–ICU^49^ scores in the medical chart and consulting the responsible ICU nurse immediately before the start of data collection. If the delirium screening was negative, participants received instructions on using the 0–10 FPT for pain intensity and the 0–10 NRS for procedural pain distress.

The NOL device was installed at the bedside, the finger probe with sensors was placed on the participant’s finger, and the device was calibrated, which took less than 5 min. To minimize potential bias, the NOL device screen was positioned so that it was not visible to the patient or any family members in the room. For participants who consented to be video recorded, the research trainee set up a video camera on a tripod at the foot of the bed to record the participant’s face and upper body, allowing for the examination of interrater reliability of CPOT scores by another trained research staff member who was not at the bedside. NOL and HR as well as pain variables were captured before, during, and 15 min after a nonnociceptive procedure (NIBP using cuff inflation) and a nociceptive procedure (suctioning; i.e., 2 procedures × 3 time points; see Table 1). The 15-min postprocedure assessment was chosen because it is estimated as the required time for the liberation, reaction, and elimination of stress hormones (epinephrine and norepinephrine).^50^

**Table 1.** Time points and variables in each group (A and B).

	Nonnociceptive procedure: Blood pressure cuff inflation			Nociceptive procedure: Suctioning		
Variable	Pre	During	Post	Pre	During	Post
NOL	A, B	A, B	A, B	A, B	A, B	A, B
CPOT	A, B	A, B	A, B	A, B	A, B	A, B
Pain intensity	A	A	A	A	A	A
Procedural pain distress	—	—	—	—	A	—

A. Patient participants able to self-report and to exhibit behaviors. B. Patient participants unable to self-report but can exhibit behaviors.

The nonnociceptive procedure involved NIBP measurement using cuff inflation and was performed first whenever possible. In cases when a patient participant required suctioning immediately, that procedure was observed first, and the NIBP measurement followed at least 15 min later. The NIBP procedure was performed by the ICU nurse or the research trainee under the ICU nurse’s supervision. Although some sensory activation may occur, this procedure was selected because it was reported as painless by ICU patients^41^ and was utilized in our pilot studies with similar findings. The nociceptive procedure comprised mouth, endotracheal, or tracheal suctioning. Suctioning procedures in mechanically ventilated ICU patients are known to cause moderate to severe pain and are also described as distressful by patients.^4–6,44,51,52^ Suctioning was performed by the ICU nurse or the respiratory therapist as part of standard care. The NOL was continuously monitored during both procedures. The research trainee manually identified the time points (i.e., start and end of the NIBP and suctioning procedures, pre/post both procedures) on the NOL device for data extraction purposes. Bedside data collection was performed in a single day.

At each time point (before, during, and 15 min after each procedure, nonnociceptive and nociceptive) CPOT scores were obtained for all participants (groups A and B). In group A, the CPOT assessment was done before obtaining the patient’s self-report of pain to minimize the rater’s bias. For participants who agreed to be video recorded, a video was taken for the duration of the suctioning procedure (i.e., less than 2 min) so a trained research staff (i.e., undergraduate nursing student) could provide CPOT scores later for interrater reliability purposes. For those who did not provide consent to be video recorded, a second trained research staff from different health-related disciplines (i.e., medicine, public health) and with significant experience with the use of the CPOT (10 and 2 years, respectively) was present at the bedside and independently performed the CPOT assessment.

After completing CPOT assessment, the research trainee asked group A participants to indicate whether they were in pain or not by either head nodding^53^ or pointing at the 0–10 FPT. Then, they were asked to rate their pain intensity. Participants who indicated no pain were assigned a score of 0. The self-reported pain intensity was not video recorded to reduce potential bias for the second CPOT rater who watched the videos later. Group A participants were asked to rate their procedural pain distress at the end of the suctioning procedure only. Finally, demographic and clinical data from the patient’s medical chart were collected for both groups. Additionally, a log was kept during data collection procedures to document any encountered problems with NOL use and the strategies employed to overcome these issues.

## Data analysis

Data analysis was performed using SPSS v27. IBM SPSS Statistics. Version 27. Descriptive statistics were computed to summarize the sociodemographic and clinical information of the study sample.

NOL data were extracted in a standardized manner, consistent with methods used in anesthesia^26,27^ and our pilot studies.^30,31^ Time points were determined from the NOL device output (e.g., before, during, and 15 min after both nonnociceptive and nociceptive procedures). Specifically, NOL values were averaged over 1-min periods at rest (before) and 15 min postprocedure. For the NIBP procedure, NOL values were averaged within a 15-s window surrounding the peak value, compromising three values before the peak, the peak value itself and three values after the peak (seven values in total). Similarly, during suctioning, NOL values were averaged within 15s surrounding the peak value obtained during the procedure. HR data, extracted from the NOL device, were processed using the same method.

Supplementary File 1 summarizes the data analysis plan. The same statistical tests were performed for the NOL (primary variable) and HR (secondary variable). Nonparametric tests were used because most variables (CPOT, pain intensity, and procedural pain distress) are considered to be ordinal, and all variables were not normally distributed at most time points (Shapiro-Wilk test, *P* < 0.05 and kurtosis and skewness indices > ±2).^54^ The alpha probability error was adjusted to 0.017 (0.05/3) for post hoc Mann-Whitney *U* and Wilcoxon signed-rank tests to account for multiple tests based on Bonferroni correction.^55^ However, when only one test was required for the research objectives, the alpha remained set at 0.05.

### Sample size

The sample size was estimated to achieve discriminative and criterion validation. For discriminative validation, we performed Friedman tests for repeated measures with all six time points (i.e., before, during, and 15 min after a nonnociceptive procedure and a nociceptive procedure). Thus, a sample of 38 participants, 19 in group A (conscious) and 19 in group B (unconscious), was estimated with an alpha of 0.05 and a power of 80%. Subsequently, Wilcoxon signed-rank tests were performed to compare pairs of time points (i.e., nociceptive and nonnociceptive procedures). A sample of 20 participants per group was deemed adequate for this analysis. Statistical analysis was set at an alpha of 0.017 for Wilcoxon signed-rank tests (Bonferroni correction) and a power of 80%, considering an effect size of 0.8 based on our previous pilot studies.^30,31^ For criterion validation, we used the Mann-Whitney *U* test using reference and alternative standard cutoff values distinguishing participants with significant pain (pain intensity ≥ 4/10 and/or CPOT ≥ 3/8) from those without significant pain (pain intensity < 4/10 and/or CPOT < 3/8).^39,56,57^ Based on combined pilot data, we used ratios of 0.5 for pain intensity and 0.7 for CPOT for participants with and without significant pain.^30,31^ Therefore, samples of 28 and 26 participants were estimated for group A and group B, respectively. Statistical analysis for Mann-Whitney *U* tests was set at an alpha of 0.05 and a power of 80%, with an effect size of 1.2, estimated from our pilot data.^30,31^ Thus, a total sample of 54 participants was estimated. Calculations were performed using G*Power 3.1.^58^

## Results

**Figure 1.**
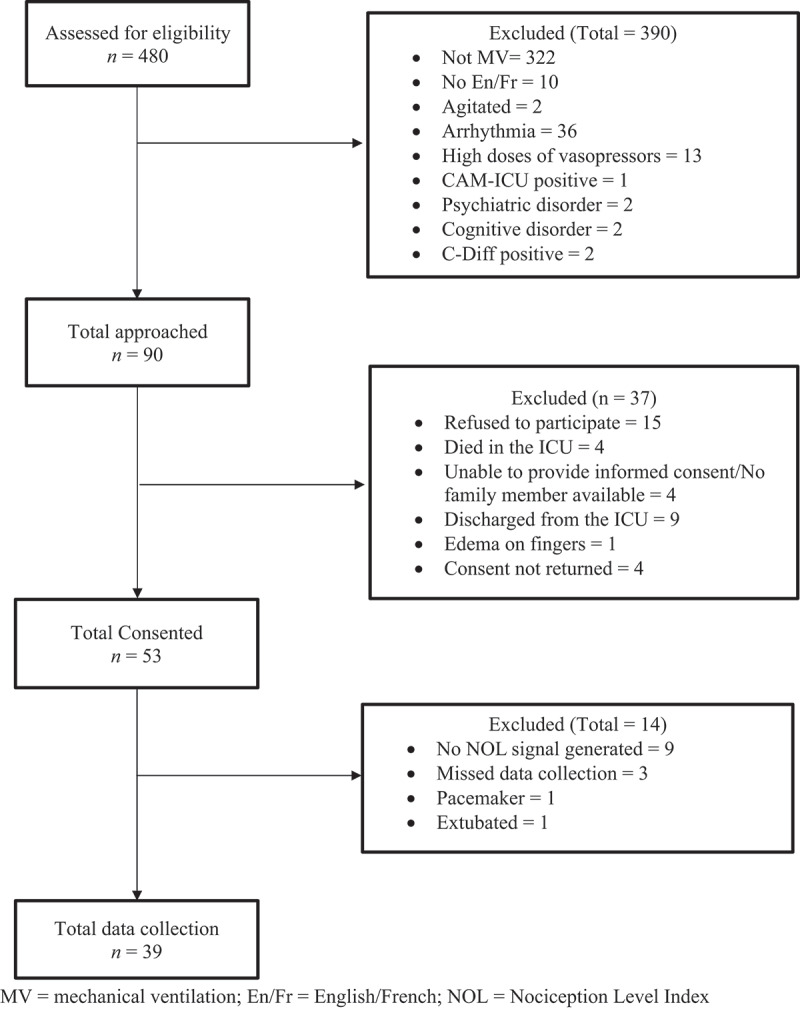
Participant Flow Diagram. MV = Mechanical ventilation; En/Fr = English/French; NOL = Nociception Level Index.

A total of 53 ICU patients were recruited over a 9-month study period (Figure 1). Data collection and analysis were completed for 39 patients (Table 2). Most participants were male and identified as men of North American and European origins, with a mean age of 64 years. All participants recorded male at birth identified as men and all participants recorded female at birth identified as women. In group B, gender was identified by a family member. Both medical (e.g., pneumonia, respiratory failure, acute kidney injury) and surgical (e.g., coronary artery disease, hemorrhagic stroke, cancer) diagnoses were represented. Sociodemographic and clinical characteristics of participants from group A and group B were similar, except for LOC and sedation levels, which were expected as per the a priori group assignment.

**Table 2.** Sociodemographic and clinical data for participating patients (*n* = 39).

Variable	Group A (*n* = 18)	Group B (*n* = 21)	Total (*n* = 39)
Age, mean (standard deviation)	63.5 (11.80)	64.4 (10.67)	64 (11.07)
Richmond Agitation Sedation Scale, median (range)	0 (0–0)	−2 (−1 to −5)	−1 (−5 to 0)
Sex and gender, *n* (%)			
Male, man	10 (55.6)	17 (81)	27 (69.2)
Female, woman	8 (44.4)	4 (19)	12 (30.8)
Ethnicity, *n* (%)			
North American origin	3 (16.7)	8 (38.1)	11 (28.2)
European origin	12 (66.7)	6 (28.6)	18 (46.2)
Caribbean origin	1 (5.6)	1 (4.8)	2 (5.1)
African origin	1 (5.6)	2 (9.5)	3 (7.7)
Asian origin	1 (5.6)	2 (9.5)	3 (7.7)
Other	0	2 (9.5)	2 (5.1)
Primary language, *n* (%)			
French	11 (61.1)	11 (52.4)	22 (56.4)
English	6 (33.3)	5 (23.8)	11 (28.2)
Other	1 (5.6)	5 (23.8)	6 (15.4)
Education level, *n* (%)			
Elementary school	0 (0)	4 (19)	4 (10.3)
Secondary (high school)	6 (33.3)	4 (19)	10 (25.6)
College	5 (27.8)	6 (28.6)	11 (28.2)
University	5 (27.8)	6 (28.6)	11 (28.2)
Missing	2 (11.1)	1 (4.8)	3 (7.7)
APACHE II, mean (standard deviation)	16.77 (10.66)	18.42 (9.05)	17.66 (9.73)
Diagnosis, *n* (%)			
Medical	8 (44.4)	7 (33.3)	15 (38.5)
Surgical	10 (55.6)	14 (66.7)	24 (61.5)
Analgesics within 4 h of suctioning procedures, *n* (%)			
Yes	11 (61.1)	13 (61.9)	24 (61.5)
No	7 (38.9)	8 (38.1)	15 (38.5)
Opioids within 4 h of suctioning procedures, morphine-equivalents per os (in mg), median (IQR)	4 (2.2–4.5)	5 (3–6)	4 (2–6)
Sedatives within 4 h of suctioning procedures, *n* (%)			
Yes	10 (55.6)	15 (71.4)	25 (64)
No	8 (44.4)	6 (28.6)	14 (36)
Vasopressors within 4 h of suctioning procedures, *n* (%)			
Yes	7 (38.9)	12 (57.1)	19 (49)
No	11 (61.1)	9 (42.9)	20 (51)
Neuromuscular blocking agent within 4 h of suctioning procedures, *n* (%)			
Yes	0	2 (9.5)	2 (5)
No	18 (100)	19 (90.5)	37 (95)

Out of 60% of participants who received analgesics within 4 h of the suctioning procedure, 92% received opioids. The median milligram doses of opioids in oral morphine-equivalents was higher in group B compared to group A (Table 2). However, the proportion of participants who received opioids did not differ by group (χ^2^ [1, *N* = 39] = 0.003, *P* = 0.959). Five participants received acetaminophen (Tylenol) at a dose of 1000 mg (one from group A) and three received ketorolac (Toradol) at a dose of 10 mg (including one from group A), in addition to opioids. From the total sample, 64% of participants received sedatives (propofol, *n* =10; Precedex, *n* =15), and higher doses were used in group B (group A = 0.02-10 mcg/kg/min vs. group B = 0.06–100 mcg/kg/min). Two participants from group B were still under the effects of neuromuscular blocking agent (rocuronium) upon their arrival to the ICU from the operating room, yet they were able to express behaviors. Additionally, 50% of participants of the total sample received low doses of vasopressors (range = 0.5–15 mcg/min; Levophed, *n* =19; concomitant adrenalin, *n* =1; Dobutrex, *n* =1; vasopressin, *n* =1).

### Description of variables before, during, and after procedures

Table 3 describes the distribution of the values of variables across time points. NOL, pain intensity, and CPOT were at their lowest values before, during, and after NIBP and before and after the suctioning procedure in both groups (A and B). Significant pain was reached during suctioning, with moderate median values: NOL > 22, pain intensity > 3, and CPOT scores > 3. HR remained stable before, during, and after NIBP and increased slightly during the suctioning procedure compared to before the suctioning and NIBP procedure (1 and 5 bpm, respectively).

**Table 3.** Medians and IQRs and discriminative ability of the NOL, self-reported pain intensity, CPOT, and HR at different time points.

	Group	*n*	Before NIBP (T1)	*n*	During NIBP (T2)	*n*	After NIBP (T3)	*n*	Before suctioning (T4)	*n*	During suctioning (T5)	*n*	After suctioning (T6)	Friedman test^a^
NOL	A	18	5.92 (3.33–12.12)	18	11.85 (4.92–21.71)	17	7.50 (4.0–17.37)	16	8.37 (3.50–17.35)	18	29.43 (21.60–43.96)	16	7.75 (3.77–14.54)	32.09*
			T1 < T2, *Z* = −2.42***				T3 < T2, *Z* = −1.63^NS^		T4 < T5, *Z* = −3.46*		T5 > T2, *Z* = −3.51*		T6 < T5, *Z* = −3.46*	
	B	21	3.08 (1.0–8.3)	21	6.85 (2.78–12.28)	18	2.36 (1.0–8.22)	20	5.50 (3.51–9.57)	21	22.14 (17.35–37.33)	19	4.50 (1.57–9.25)	35.14*
			T1 < T2, *Z* = −2.48***				T3 < T2, *Z* = −1.54^NS^		T4 < T5, *Z* = −3.66*		T5 > T2, *Z* = −3.84*		T6 < T5, *Z* = −3.70*	
	Total	39	4.25 (1.08–9.0)	39	9.29 (4.29–14.29)	35	5.08 (1.67–10.33)	36	6.71 (3.51–12.06)	39	26.30 (17.57–41.57)	35	5.83 (2.33–10.67)	64.28*
			T1 < T2, *Z* = −3.45*				T3 < T2, *Z* = −2.09***		T4 < T5, *Z* = −5.04*		T5 > T2, *Z* = −5.19*		T6 < T5, *Z* = −5.05*	
Pain intensity	A	17	0 (0–0.50)	18	0 (0–0)	17	0 (0–0)	16	0 (0–0)	18	3 (0–6.2)	16	0 (0–0.75)	18.70***
			*Z* = −0.82^NS^				*Z* = −0.45^NS^		T4 < T5, *Z* = −2.60***		T5 > T2, *Z* = −2.63**		T6 < T5, *Z* = −2.80***	
CPOT	A	18	0 (0–0)	18	0 (0–0)	17	0 (0–0)	16	0 (0–0)	18	3 (2.0–4.25)	16	0 (0–0)	60.71*
			*Z* = −1.41^NS^				*Z* = −1.63^NS^		T4 < T5, *Z* = −3.53*		T5 > T2, *Z* = −.64*		T6 < T5, *Z* = −3.43*	
	B	20	0 (0–0)	20	0 (0–0)	17	0 (0–0)	19	0 (0–0)	21	3 (2.0–4.0)	18	0 (0–0.25)	39.91*
			*Z* = −1.41^NS^				*Z* = −1.14^NS^		T4 < T5, *Z* = −3.54*		T5 > T2, *Z* = −.75*		T6 < T5, *Z* = −3.20*	
	Total	38	0 (0–0)	38	0 (0–0)	34	0 (0–0)	35	0 (0–0)	39	3 (2.0–4.0)	34	0 (0–0)	96.80*
			*Z* = −2.00***				*Z* = −0.26^NS^		T4 < T5, *Z* = −4.97*		T5 > T2, *Z* = −5.19*		T6 < T5, *Z* = −4.65*	
HR	A	18	83.70 (71.14–93.39)	18	82.00 (71.71–92.29)	17	84.50 (70.08–94.87)	16	82.29 (76.39–90.45)	18	88.43 (77.56–96.54)	16	82.16 (76.35–93.37)	11.81***
			*Z* = −0.66^NS^				*Z* = −1.91^NS^		T4 < T5, *Z* = −2.22***		T5 > T2, *Z* = −2.90**		*Z* = −1.87^NS^	
	B	21	72.08 (65.58–87.50)	21	71.43 (67.78–85.71)	18	70.54 (64.64–87.43)	20	75.36 (69.04–89.46)	21	75.71 (69.57–91.43)	19	76.83 (69.50–87.25)	22.90*
			*Z* = −0.37^NS^				*Z* = −0.69^NS^		*Z* = −1.87^NS^		T5 > T2, *Z* = −2.49***		*Z* = −1.65^NS^	
	Total	39	78.50 (68.67–89.58)	39	78.57 (68.14–91.71)	35	76.33 (68.08–91.83)	36	77.83 (70.31–90.02)	39	82.29 (72.00–93.00)	35	78.33 (70.00–89.83)	31.03*
			*Z* = −0.35^NS^				*Z* = −1.05^NS^		T4 < T5, *Z* = −2.81**		T5 > T2, *Z* = −3.78*		T6 < T5, *Z* = −2.83**	

*Notes*. Outcomes are reported as median (IQR). Differences in sample sizes result from missing data across study time points.

^a^Friedman’s tests were conducted on a smaller sample due to missing data across study time points.

*P ≤ 0.001, **P < 0.01, ***P < 0.05, ^NS^not significant.

### Discriminative validation

Friedman tests among repeated measures were significant for all variables in the total sample and each group (Table 3). Subsequently, Wilcoxon signed-rank test (*Z*) showed that NOL and HR values were significantly higher during suctioning (i.e., nociceptive procedure) compared to NIBP (i.e., nonnociceptive procedure; Table 3).

No significant differences in NOL and HR median values between group A and group B were found during the suctioning procedure (*U* = 152.00, *P* = 0.297; *U* = 120.50, *P* = 0.054, respectively; Table 3). Complementary analyses also indicated no significant differences in CPOT scores between group A and group B during the suctioning procedure (*U* = 178.50, *P* = 0.763; Table 3). Therefore, NOL, HR, and CPOT did not differ according to the level of consciousness (group A and group B).

### Criterion validation

From the 18 conscious participants from group A, seven denied experiencing pain (39%), and the other 11 reported a median of 5 (interquartile range [IQR] = 4–7) on a 0–10 FPT during the suctioning procedure. NOL values were higher in participants with significant pain intensity ≥ 4/10 (*n* = 9, median = 41.57, IQR =30–46.14) than those without significant pain intensity < 4/10 (*n* = 9, median = 23, IQR = 15.07–28.85, *U* = 13.00, *P* < 0.05). However, HR values remained similar between participants with significant pain and those without significant pain (*U* = 20.00, *P* = 0.07).

For CPOT scores during the suctioning procedure, interrater reliability between two raters (i.e., bedside rater and trained research staff using videos [*n* =30] or a second bedside rater [*n* =9]) was supported with an ICC = 0.89 (95% confidence interval 0.80–0.95, *P* < 0.001). Considering high interrater reliability, CPOT scores from the bedside rater were used for data analysis. In group A, NOL values were higher in participants with CPOT scores ≥ 3/8, indicating significant pain (*n* = 10, median = 37.28, IQR = 27.57–46.32) than in those without significant pain (CPOT < 3/8; *n* = 8, median = 24.35, IQR = 13.60–29.82, *U* = 13.00, *P* < 0.05). However, HR values remained similar between participants with significant pain and those without significant pain according to CPOT scores (*U* = 29.50, *P* = 0.36). In group B, NOL values were similar between participants with CPOT scores indicating significant pain (*n* = 12, median = 23.51, IQR = 20.85–37.81) and those without significant pain (*n* = 9, median = 20.30, IQR = 12.14–36.21, *U* = 39.50, *P* = 0.31). Similarly, HR values did not differ between participants with significant pain and those without significant pain according to CPOT scores (*U* = 50.00, *P* = 0.808). In the total sample, NOL values were higher in participants with CPOT scores indicating significant pain (*n* = 22, median = 30.71, IQR = 21.93–46.07) than in those without significant pain (*n* = 17, median = 23, IQR = 13.78–29.64, *U* = 114.50, *P* < 0.05). However, HR values did not differ between participants with significant pain and those without significant pain according to CPOT scores (*U* = 339.00, *P* = 0.977).

Positive correlations were found between the NOL and pain standard measures. In group A, a moderate positive correlation was obtained with self-reported pain intensity (ρ = 0.65, *P* < 0.05) but a nonsignificant moderate positive correlation was found with CPOT (ρ = 0.40, *P* = 0.103; Figures 2a,b) In group B, a nonsignificant weak positive correlation was found with CPOT (ρ = 0.31, *P* = 0.312; Figure 2e). In the total sample, a weak positive correlation was found between NOL and CPOT (ρ = 0.32, *P* < 0.05).

For HR, nonsignificant weak negative correlations were found with pain intensity (ρ = −0.39, *P* = 0.114) and with CPOT (ρ = −0.28, *P* = 0.259) in group A (Figure 2c,d). No correlation was found between HR and CPOT in group B (ρ = 0.02, *P* = 0.168; Figure 2f), as well as in total sample (ρ = −0.05, *P* = 0.746).

### Convergent validation

Of the 16 conscious participants in group A who rated their procedural pain distress level on 0–10 NRS, 5 denied experiencing distress, and the other 11 reported a median of 7 (IQR = 5–9) during the suctioning procedure. No differences were found between NOL or HR values of patients with a significant distress level ≥ 4/10 (*n* = 10) and those without a significant distress level (<4/10; *n* = 6; NOL: *U* = 29.00, *P* = 0.914; HR: *U* = 24.00, *P* = 0.515). A nonsignificant weak negative correlation was found between NOL and distress level (ρ = −0.16, *P* = 0.565), and no correlation was found between HR and distress level (ρ = −0.06, *P* = 0.817; Figures 3a,b).

**Figure 2.**
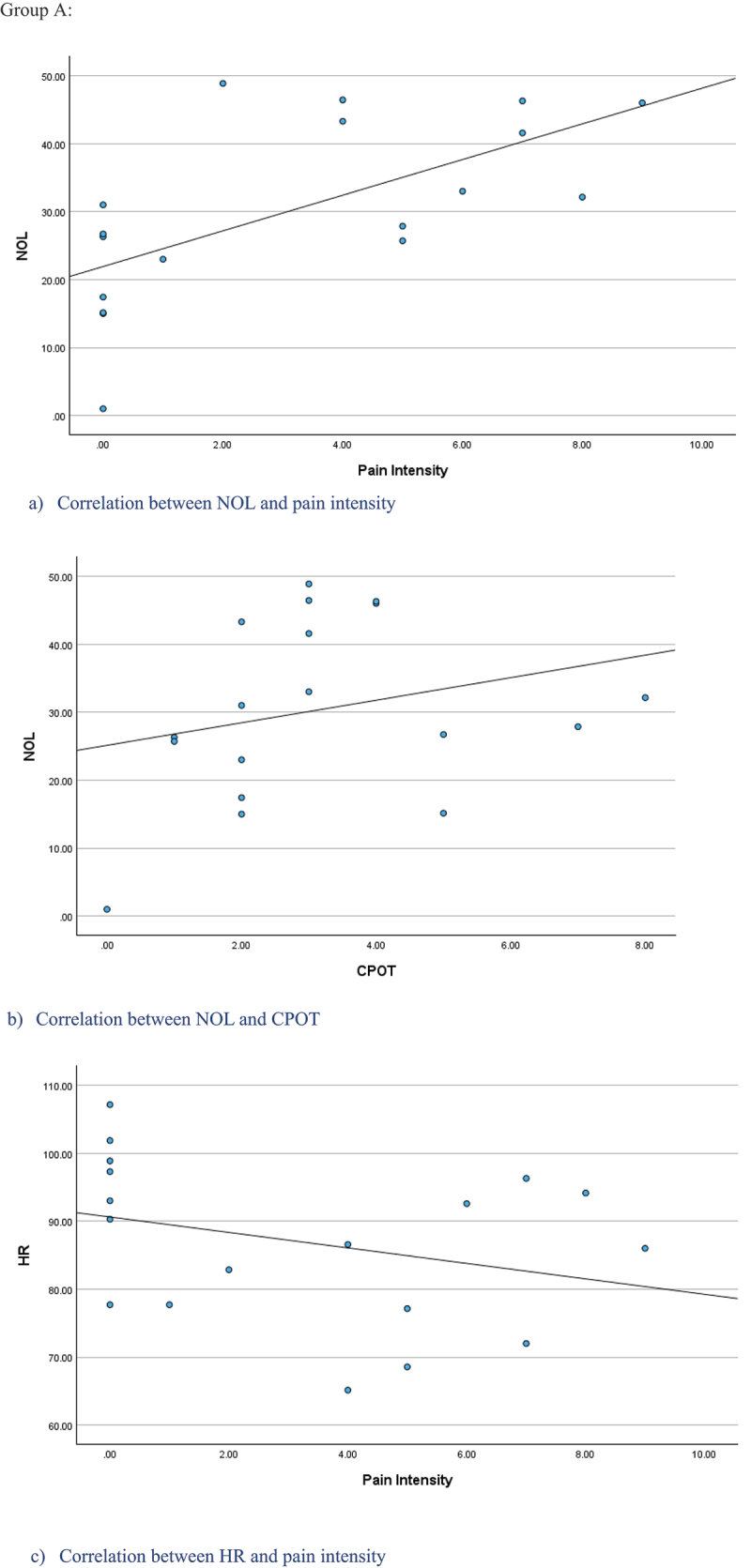
Scatter plots of the NOL, self-reported pain intensity, CPOT, and HR during suctioning procedures for Group A and B.

**Figure 2.**
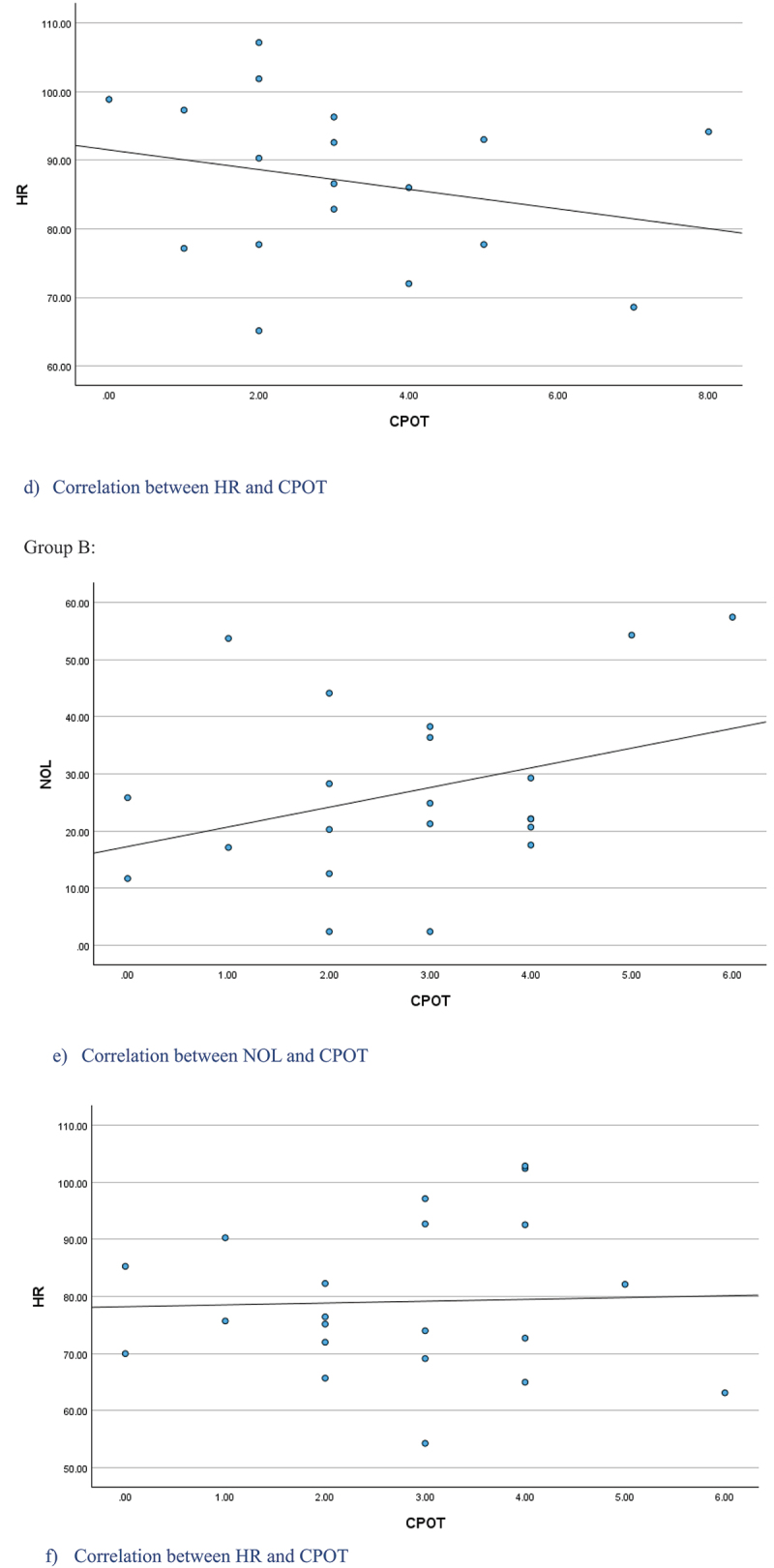
(Continued).

### Test–retest reliability

Fifteen minutes after the suctioning procedure, NOL values reached similar values as before the procedure for both group A (*Z* = −1.04, *P* = 0.30) and group B (*Z* = −0.14, *P* = 0.887). HR values also remained similar before and after procedure in each group (group A: *Z* = −0.38, *P* = 0.706; group B: *Z* = −0.41, *P* = 0.679). Similar findings were found in the total sample for NOL (*Z* = −0.91, *P* = 0.362) and HR values (*Z* = −0.10, *P* = 0.918).

### Feasibility of research methods

A total of 480 ICU patients were screened for eligibility over a 9-month study period (Figure 1). Of these, 390 patients were not eligible mainly because they were not mechanically ventilated, having cardiac arrhythmias (e.g., atrial fibrillation, multiform PVCs, frequent SVBPs), or receiving high doses of vasopressors. Of those who were eligible and approached, 15 refused to participate (i.e., 2 patients and 13 family members), indicating feeling overwhelmed and anxious with their condition and ICU admission. In total, 22 patients were excluded mainly due to ICU discharge, death, or not returning the signed consent form.

**Figure 3.**
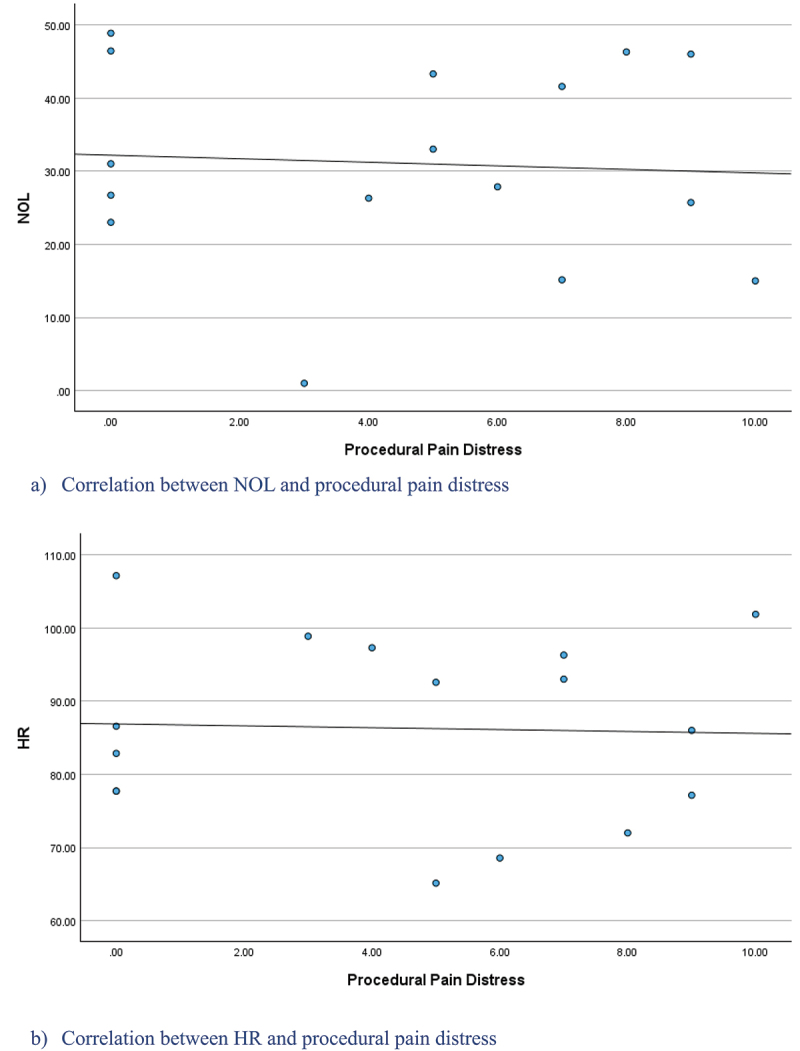
Scatter plots of the NOL, HR, and procedural pain distress during suctioning procedures for Group A.

Of the 53 enrolled patients (11% of ICU patients screened), the NOL signal did not generate in 9 of them. Rapid changes in clinical condition (e.g., arrhythmias) during data collection led to the NOL signal either not being generated or being lost. Data collection was missed in three participants because the suctioning procedure happened during the night shift when no research staff were available. Two participants were excluded because one provided their consent after extubation and the other required the installation of a pacemaker and became ineligible. Consequently, the final sample size included in data analysis was 39 patients, representing 43% of eligible participants and an attrition rate of 26%. A main challenge in the recruitment process included multiple ICU visits to engage with patients or their families, who were often overwhelmed by their loved ones’ critical condition. Mitigation strategies involved scheduled follow-ups and close collaboration with ICU nurses to notify the research staff when a family member was present.

Several participants developed complications such as edema, cold extremities, and dehydrated skin due to their critical condition, leading to NOL data collection challenges. Considering that the NOL finger probe is available in a one-size format, we devised a solution for participants with edema or large fingers by folding the upper part of the finger probe and securing it with additional tape (*n* = 4), which reduced pressure on swollen fingers. Another solution was to relocate the probe to another finger during data collection (*n* = 1). Nevertheless, in cases of severe edema (*n* = 3), neither of these mitigation strategies yielded favorable results. For participants with cold fingers (*n* = 3), we wrapped the hand in a warm blanket or performed a light-pressure hand massage to obtain optimal signal quality. For those with dry skin (*n* = 2), we applied a water-soluble lubricant to the skin before placing the finger probe, which facilitated the generation of the NOL signal.

## Discussion

This validation study of the NOL was conducted in a heterogeneous sample of mechanically ventilated ICU patients at different LOCs. Our findings supported that the NOL captures nociception in critically ill adults. In both groups, the NOL could discriminate between nonnociceptive and nociceptive procedures (i.e., nociception as part of physiologic dimension of pain). NOL values were stable at rest pre- and postsuctioning, supporting test–retest reliability. The NOL correlated moderately with pain intensity (i.e., sensory dimension of pain) but weakly with CPOT scores (i.e., behavioral dimension of pain). Though an association with CPOT scores was observed in conscious patients (group A), this relationship was not statistically significant in those with an altered LOC (group B) due to our small sample size, which affected statistical power. Furthermore, the NOL was not associated with procedural pain distress (i.e., emotional dimension of pain). Potential misunderstanding of the distress question was flagged for some participants. As anticipated, the validity of HR for ICU nociception and pain assessment was not supported. Though not all dimensions of pain must be present for the pain experience to occur, these findings reinforce the complexity and multidimensional nature of pain and highlight the importance of using assessment methods that are adapted to the patient’s ability to communicate and clinical condition.

Discriminative validity findings were consistent with those of our pilot studies in which NOL values (medians >25) were higher during endotracheal suctioning^30^ and chest tube removal^31^ compared to rest (i.e., pre and post procedures) and NIBP. Our findings were also consistent with a recent pilot validation study in 18 sedated mechanically ventilated ICU patients in which NOL values were higher (mean >32) during endotracheal suctioning compared to baseline/rest values.^59^ Comparable NOL discriminative findings were reported in anesthetized mechanically ventilated patients undergoing surgery,^29^ with NOL median increases ranging from 16 to 39 after nociceptive stimuli (e.g., intubation, skin incision) or standardized tetanic stimulation compared to no stimulation or rest periods.^23–27,60–62^

In this study, HR also increased during suctioning procedures by 5% compared to rest presuctioning, but these changes were not clinically significant (>20%).^1^ These findings are consistent with our narrative review reporting that HR did not reach clinical significance in 90% of the 30 included discriminative validation studies.^21^ Nociceptive procedures such as suctioning may trigger both nociception and the biological stress response, leading to the release of catecholamines such as epinephrine. The chronotropic effects of epinephrine on cardiac activation can manifest as increased HR.^63^

Criterion validity of the NOL was supported with the reference standard measure of pain (i.e., self-reporting). Higher NOL values were observed in conscious patients (group A) who reported significant pain intensity compared to those who reported no significant pain during suctioning procedures.^30^ Furthermore, the NOL generated stronger correlations with self-reported pain intensity compared to HR, which did not correlate with this reference standard pain measure, as also found in previous ICU studies.^21^

Criterion validity of the NOL using an alternative standard pain measure (i.e., CPOT) led to inconsistent findings in the two groups. Conscious patients (group A) with significant pain according to CPOT scores had higher NOL values compared to those with no significant pain. These findings were similar to those found in our previous pilot study.^30^ Also, a moderate correlation with a tendency toward significance was observed between the NOL and CPOT in group A. In a recent pilot randomized controlled trial, mild to strong correlations were found between the NOL and both self-reported pain intensity and CPOT scores during pain assessments at rest in 30 conscious surgical ICU patients.^64^ However, in group B, NOL values did not differ according to CPOT scores and the correlation between NOL and CPOT did not reach statistical significance. Bonvecchio et al.^59^ also reported a nonsignificant weak correlation (*r* = 0.12; *P* = 0.363) between the NOL and the Behavioral Pain Scale (BPS) during endotracheal suctioning in deeply sedated patients unable to self-report. It is worth highlighting that the applicability of behavioral pain measures is questionable in deeply sedated patients because responses to stimulation may be blurred by the medication. Although not statistically significant, correlations between the NOL and CPOT were higher than those found between HR and CPOT. This is also aligned with previous studies that do not support criterion validity between HR and behavioral pain measures in critically ill adults.^21^ Further validation of the NOL with behavioral pain measures is required in larger samples of patients with an altered LOC and responsive to stimulation.

Nonsignificant negative correlations were observed between HR and pain standard measures. Two other studies have also reported negative correlations between HR and CPOT during suctioning.^65,66^ This procedure typically stimulates the cough reflex and activates the sympathetic nervous system, which is known to lead to HR increases. However, it may also trigger vagal parasympathetic responses that result in HR decreases. The hemodynamic changes observed during suctioning likely reflect the interplay between these opposing autonomic responses.^67^

Convergent validity of the NOL with procedural pain distress was examined for the first time but was not supported. We expected participants to report distress related to the suctioning procedures, reflecting one aspect of the emotional dimension of pain.^42^ It is possible that all participants did not sufficiently understand the question regarding procedural pain distress, leading to unreliable self-reports. In the pilot study with cardiac surgery ICU patients, a weak association was found between NOL > 25 and pain unpleasantness,^31^ a well-established indicator of the emotional dimension of pain.^68^ Similar to NOL, convergent validity of HR was not supported.

Regarding the feasibility of the research methods, 74% of consented participants were included in data analysis, and the NOL signal did not generate in nine of them (17%). Similar challenges, such as cold fingers and dry skin, with low-quality or absent NOL signals, were reported in previous studies.^30,31,59^ To mitigate these challenges, we used similar strategies across studies such as wrapping the hand holding the probe with a warm towel and lubricating the skin before installing the probe.^30,31^ In addition, we found that edema could cause signal loss. Given the complexity and heterogeneity of this population, identifying all relevant exclusion criteria during study planning is challenging, with some issues only becoming apparent after patient inclusion begins.^69^ When appropriate, certain issues may be addressed by adjusting eligibility criteria.

### Strengths and limitations

The strengths of this study include the use of validation strategies to capture the multidimensionality of pain. Most important, ICU pain standard measures, including self-reporting and CPOT, were obtained. Other validity testing using emotional dimension (i.e., procedural pain distress) could have broadened the validation outcomes; however, the reliability of the procedural pain distress findings was questionable.

Our study sample included only mechanically ventilated ICU patients without severe cardiovascular conditions (e.g., arrhythmias) to improve internal validity. Additionally, our small sample size from a single site limited the external validity of these study findings.

We experienced missing data at multiple time points, resulting in a variable sample size for our objectives. We decided not to replace the missing data because they occurred due to missed observations unrelated to the data values (i.e., missing at random).^70^ For example, in 16 patients, the nociceptive procedure occurred first because the patient participant required it immediately. As a result, the pre or post time points were missed or the NIBP procedure was recorded afterward. The NOL effect size in this study was 1.8, slightly larger than that of our previous pilot studies. Therefore, our sample size led to sufficient power for most primary analyses. Of note, the sample size estimation did not account for attrition due to timeline constraints. The attrition rate was 26%, lower than the 45% reported in both pilot studies.^30,31^

A CPOT rating bias at the bedside remained possible, because the rater was not blinded to the nociceptive procedure and could anticipate more intense behaviors. Yet, we addressed this potential bias by the examination of interrater reliability between two CPOT raters. A high ICC confirmed excellent interrater reliability between CPOT raters.^71^

We could only evaluate HR performance because it is the sole individual parameter provided by the NOL device. However, other studies have evaluated the performance of the NOL versus individual parameters (HR, BP, and skin conductance) in surgical anesthetized mechanically ventilated patients, and the NOL outperformed these individual parameters.^23–27,62,72^ These findings suggest the superiority of a multiparametric approach to individual parameters in this population. Future research could compare individual parameters (e.g., vital signs, skin conductance) with the NOL for nociception and pain assessment in ICU patients.

Another potential limitation of this study is the lack of data collection regarding participants’ prior pain experiences, such as a history of chronic pain or previous use of analgesics. These factors may influence both behavioral pain expression and physiological responses to nociceptive stimuli. For instance, individuals with chronic pain often exhibit altered autonomic regulation, including reduced heart rate variability, which may affect the NOL’s output.^73^ Future studies should consider collecting and controlling for prior pain history in critically ill patients.

Finally, the clinician performing the suctioning procedure may have influenced the patient’s experience of pain or discomfort. Variability in the suctioning technique could have affected how patients perceived and reported procedural pain. However, because data on individual clinicians were not collected, it was not possible to examine the extent of this potential influence. This variability may have contributed to differences in self-reports of pain among conscious patients and should be taken into account in the interpretation of the findings.

## Conclusion

The NOL could discriminate between nociceptive and nonnociceptive procedures as part of ICU standard care and showed stable values at rest pre and post nociceptive procedure. Criterion validity of the NOL with pain standard measures (self-report and CPOT) was supported in conscious patients (group A) but did not reach statistical significance in those with an altered LOC (group B). The NOL did not correlate with self-reported procedural pain distress, and convergent validity was not supported. The NOL has limited use in critically ill adults with specific conditions, including arrhythmias, shock, edema, and agitation. Further validation of the NOL in a larger and more heterogeneous sample of ICU patients is still needed.

## Supplementary Material

Supplementary File 1_NOL validation Final_Shahiri et al 2025.docx
