# Supplementary material for: Nociception and pain assessment during suctioning procedures in mechanically ventilated patients in the intensive care unit: A validation study of the Nociception Level index (NOL™)
Source: Can J Pain. 2026 Apr 29;10(1):2561576. doi: 10.1080/24740527.2025.2561576 (PMC13134404; doi:10.1080/24740527.2025.2561576)
Supplement: Supplementary File 1_NOL validation Final_Shahiri et al 2025.docx [file UCJP_A_2561576_SM9778.docx]

Supplementary File 1. Data analysis per validation objective

| Study objective | Hypothesis | Dependent and independent variables | Statistical tests |
| --- | --- | --- | --- |
| Descriptive statistics | Not applicable | Dependent variables:   - NOL - 0-10 Pain intensity - 0-8 CPOT - 0-10 Procedural pain distress | Medians and Interquartile range (IQR) |
| Discriminative Validation | NOL values were expected to be higher during the nociceptive procedure compared to the non-nociceptive procedure. | Dependent variable:   - NOL   Independent variable:   - Time points | Friedman tests: to compare NOL and pain scores^1^ before, during, and after each procedure in each group (A and B) and total sample.  Wilcoxon signed-rank tests: to compare NOL during non-nociceptive and nociceptive procedures in each group (A and B) and total sample.  Mann-Whitney U tests (complementary for group comparison): to determine whether there are any significant differences between the NOL during the nociceptive procedure in Group A (conscious) versus in Group B (altered LOC). |
| Criterion Validation | a) NOL values were expected to be higher in participants with significant pain than those without significant pain according to the appropriate pain standard measure (i.e., self-reported pain intensity or CPOT) during the nociceptive procedure.  b) NOL values were expected to be correlated with pain scores during the nociceptive procedure. | Dependent variable:   - NOL   Independent variables:   - 0-10 Pain intensity - 0-8 CPOT   For hypothesis a:   - Dichotomized Pain intensity scores:   0 - 3 = no significant pain  4 - 10 = significant pain   - Dichotomized CPOT scores:   0 - 2 = no significant pain  3 - 8 = significant pain | a) Mann-Whitney U tests: to compare NOL between participants with significant pain and without significant pain during the nociceptive procedure in each group (A and B) and total sample.  b) Spearman correlations: between NOL and pain scores in participants able to self-report (Group A) or exhibit pain-related behaviors (Group A and B).^*^ |
| Convergent Validation | a) NOL values were expected to be higher in participants with significant procedural pain distress during the nociceptive procedure.  b) NOL values were expected to be correlated with procedural pain distress. | Dependent variable:   - NOL   Independent variable:   - 0-10 Procedural pain distress   For hypothesis a:   - Dichotomized procedural pain distress scores:   0 - 3 = no significant distress  4 - 10 = significant distress | a) Mann-Whitney U tests: to compare NOL between participants with significant procedural pain distress and without significant procedural pain distress during the nociceptive procedure in Group A.  b) Spearman correlations: between NOL and self-reported procedural pain distress during the nociceptive procedure in Group A. |
| Test-retest Reliability | NOL values were expected to not differ significantly before versus after the nociceptive procedure. | Dependent variable:   - NOL   Independent variable:   - Time points | Wilcoxon Signed Rank test: to determine whether NOL differed before versus after the nociceptive procedure in each group and total sample. |

* Intraclass correlation coefficient (ICC) was calculated to assess CPOT scores between the bedside rater and another trained rater who watched the videos/second bedside rater

1 Complementary analysis was performed with pain intensity and CPOT variables.
